# Dihydro­cryptopine

**DOI:** 10.1107/S1600536812017588

**Published:** 2012-05-16

**Authors:** Wenwen Sun, Lei Cao, Cen Zhang, Lifei Zhu, Le Zhou

**Affiliations:** a College of Science, Northwest Agriculture and Forestry University, Yangling 712100, People’s Republic of China

## Abstract

In the crystal structure of the title compound [systematic name: 6,7-dimeth­oxy-12-methyl-16,18-dioxa-12-aza­tetra­cyclo­[12.7.0.0^4,9^.0^15,19^]henicosa-1(21),4,6,8,14,19-hexaen-3-ol], C_21_H_25_NO_5_, the benzene rings exhibits a dihedral angle of 14.95 (4)°. In the crystal, mol­ecules are linked by pairs of O—H⋯O hydrogen bonding into inversion dimers. These dimers are further connected by C—H⋯O inter­actions.

## Related literature

For the synthesis of the title compound, see: Wada *et al.* (2007 ▶). For the biological activity of cryptopine derivatives, see: Morteza *et al.* (2003 ▶); Yang *et al.* (2009 ▶); Capasso *et al.* (1997 ▶); Jeong *et al.* (2009 ▶).
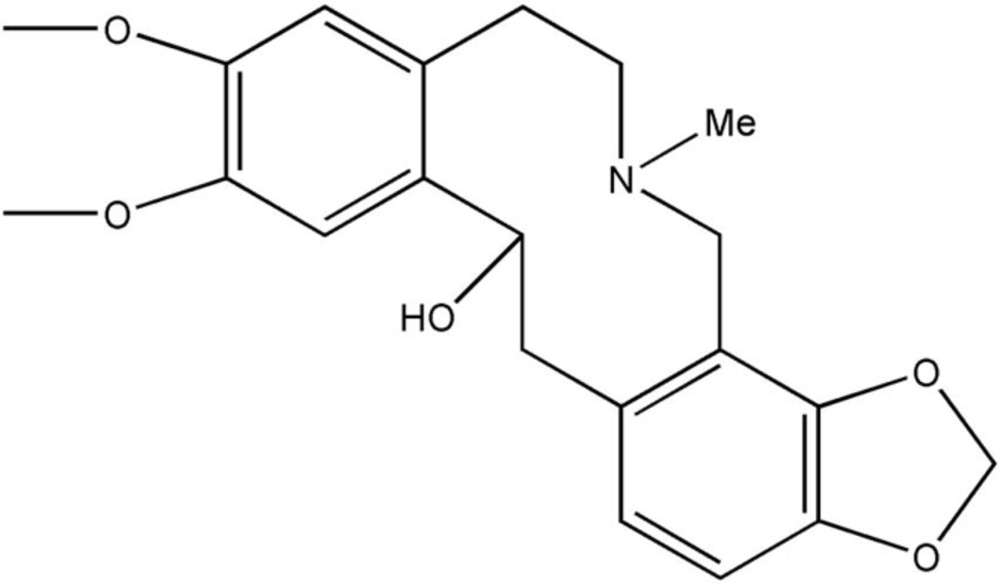


## Experimental

### 

#### Crystal data

C_21_H_25_NO_5_
*M* *_r_* = 371.42
Monoclinic, 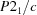

*a* = 9.5810 (16) Å
*b* = 6.7405 (12) Å
*c* = 28.886 (5) Å
β = 92.164 (2)°
*V* = 1864.2 (6) Å^3^
*Z* = 4
Mo *K*α radiation
μ = 0.09 mm^−1^
*T* = 296 K
0.26 × 0.21 × 0.18 mm

#### Data collection

Bruker SMART APEXII CCD area-detector diffractometer
Absorption correction: multi-scan (*SADABS*; Sheldrick, 1996 ▶) *T*_min_ = 0.976, *T*_max_ = 0.983
13267 measured reflections
3460 independent reflections
2721 reflections with *I* > 2σ(*I*)
*R*_int_ = 0.021

#### Refinement

*R*[*F* ^2^ > 2σ(*F* ^2^)] = 0.038
*wR*(*F* ^2^) = 0.105
*S* = 1.02
3460 reflections
248 parameters
H-atom parameters constrained
Δρ_max_ = 0.12 e Å^−3^
Δρ_min_ = −0.18 e Å^−3^

### 

Data collection: *APEX2* (Bruker, 2004 ▶); cell refinement: *SAINT* (Bruker, 2004 ▶); data reduction: *SAINT*; program(s) used to solve structure: *SHELXS97* (Sheldrick, 2008 ▶); program(s) used to refine structure: *SHELXL97* (Sheldrick, 2008 ▶); molecular graphics: *SHELXTL* (Sheldrick, 2008 ▶); software used to prepare material for publication: *SHELXTL*.

## Supplementary Material

Crystal structure: contains datablock(s) global, I. DOI: 10.1107/S1600536812017588/nc2270sup1.cif

Structure factors: contains datablock(s) I. DOI: 10.1107/S1600536812017588/nc2270Isup2.hkl

Supplementary material file. DOI: 10.1107/S1600536812017588/nc2270Isup3.cml

Additional supplementary materials:  crystallographic information; 3D view; checkCIF report

## Figures and Tables

**Table 1:** Hydrogen-bond geometry (Å, °)

*D*—H⋯*A*	*D*—H	H⋯*A*	*D*⋯*A*	*D*—H⋯*A*
O3—H3⋯O4^i^	0.82	2.31	3.0924 (16)	159
C1—H1*A*⋯O3^ii^	0.97	2.41	3.367 (2)	170

Symmetry codes: (i) 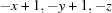; (ii) 
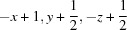
.

**Fig. 1.**
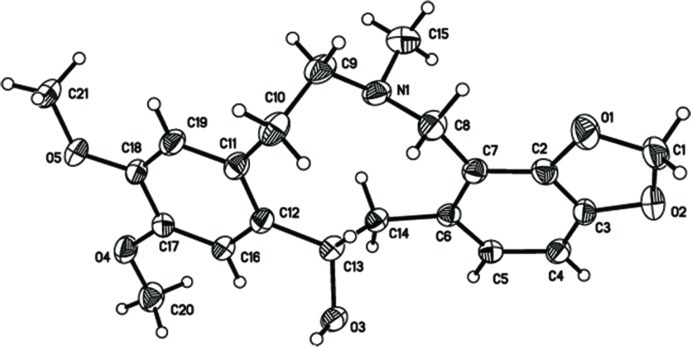
ORTEP drawing of the title compound with labeling and displacement ellipsoids drawn at the 30% probability level.

## supplementary crystallographic information

### Comment

The cryptopine derivatives have recently attracted great attention due to their antifungal (Morteza *et al.* 2003) and antibacterial activity (Yang *et al.* 2009), their analgesic effect (Capasso *et al.* 1997) and anti-dementia prperties (Jeong *et al.* 2009). In this context we are interested in the synthesis of cryptopine derivatives with biological activity. Within this project the crystal structure of the title compound was determined.

The molecule of the title compound is characterized by the presence of a ten-membered ring (hexahydrodibenzo*[c,g]*azecine) with a methylated tertiary nitrogen atom and a hydroxyl group fused to two aryl moieties (Fig. 1). In general, the title compound have two oxygenated substituents on the benzene ring and two methoxyl on the other benzene ring.Benzene rings C2/C3/C4/C5/C6/C7 and C11/C12/C16/C17/C18/C19 are inclined with respect to one another with a dihedral angle of 19.949 (41)°.

In the crystal structure, two adjacent molecules are linked by intermolecular O—H···O hydrogen bond into centrosymmetrically dimers that are further connected into layers by weak C—H···O interactions (Table 1).

### Experimental

The title compound was synthesized according to the literature procedure (Wada *et al.* 2007), and crystals were obtained from a solution in methanol by slow evaporation of the solvent at room temperature.

### Refinement

H atoms were positioned geometrically (O-H H atoms allowed to rotate but not to tip) and treated as riding, with C—H bond lengths constrained to 0.93 (aromatic CH), or 0.97 Å (methylene CH_2_), or 0.96Å (methyl CH_3_), and O—H = 0.82 Å, and with U_iso_(H) = 1.2U_eq_(C) or U_iso_(H) = 1.5U_eq_(O).

### Crystal data

**Table:**

C_21_H_25_NO_5_	*F*(000) = 792
*M**_r_* = 371.42	*D*_x_ = 1.323 Mg m^−^^3^
Monoclinic, *P*2_1_/*c*	Mo *K*α radiation, λ = 0.71073 Å
*a* = 9.5810 (16) Å	Cell parameters from 4124 reflections
*b* = 6.7405 (12) Å	θ = 2.5–26.5°
*c* = 28.886 (5) Å	µ = 0.09 mm^−^^1^
β = 92.164 (2)°	*T* = 296 K
*V* = 1864.2 (6) Å^3^	Block, colourless
*Z* = 4	0.26 × 0.21 × 0.18 mm

### Data collection

**Table:**

Bruker SMART APEXII CCD area-detector diffractometer	3460 independent reflections
Radiation source: fine-focus sealed tube	2721 reflections with *I* > 2σ(*I*)
Graphite monochromator	*R*_int_ = 0.021
phi and ω scans	θ_max_ = 25.5°, θ_min_ = 2.5°
Absorption correction: multi-scan (*SADABS*; Sheldrick, 1996)	*h* = −11→11
*T*_min_ = 0.976, *T*_max_ = 0.983	*k* = −8→8
13267 measured reflections	*l* = −34→34

### Refinement

**Table:**

Refinement on *F*^2^	Primary atom site location: structure-invariant direct methods
Least-squares matrix: full	Secondary atom site location: difference Fourier map
*R*[*F*^2^ > 2σ(*F*^2^)] = 0.038	Hydrogen site location: inferred from neighbouring sites
*wR*(*F*^2^) = 0.105	H-atom parameters constrained
*S* = 1.02	*w* = 1/[σ^2^(*F*_o_^2^) + (0.0533*P*)^2^ + 0.3327*P*] where *P* = (*F*_o_^2^ + 2*F*_c_^2^)/3
3460 reflections	(Δ/σ)_max_ < 0.001
248 parameters	Δρ_max_ = 0.12 e Å^−^^3^
0 restraints	Δρ_min_ = −0.18 e Å^−^^3^

### Special details

**Table:**

Geometry. All e.s.d.'s (except the e.s.d. in the dihedral angle between two l.s. planes)are estimated using the full covariance matrix. The cell e.s.d.'s are takeninto account individually in the estimation of e.s.d.'s in distances, anglesand torsion angles; correlations between e.s.d.'s in cell parameters are onlyused when they are defined by crystal symmetry. An approximate (isotropic)treatment of cell e.s.d.'s is used for estimating e.s.d.'s involving l.s. planes.
Refinement. Refinement of *F*^2^ against ALL reflections. The weighted *R*-factor *wR* and goodness of fit *S* are based on *F*^2^, conventional *R*-factors *R* are based on *F*, with *F* set to zero for negative *F*^2^. The threshold expression of *F*^2^ > σ(*F*^2^) is used only for calculating *R*-factors(gt) *etc*. and is not relevant to the choice of reflections for refinement. *R*-factors based on *F*^2^ are statistically about twice as large as those based on *F*, and *R*- factors based on ALL data will be even larger.

### Fractional atomic coordinates and isotropic or equivalent isotropic displacement parameters (Å^2^)

**Table:**

	*x*	*y*	*z*	*U*_iso_*/*U*_eq_	
C1	0.7221 (2)	0.5338 (3)	0.34220 (6)	0.0678 (5)	
H1A	0.6576	0.6282	0.3550	0.081*	
H1B	0.8016	0.5203	0.3638	0.081*	
C2	0.72327 (15)	0.4630 (2)	0.26692 (5)	0.0479 (4)	
C3	0.65789 (15)	0.3100 (3)	0.28885 (5)	0.0487 (4)	
C4	0.60582 (15)	0.1506 (2)	0.26509 (5)	0.0487 (4)	
H4	0.5641	0.0445	0.2798	0.058*	
C5	0.61899 (14)	0.1562 (2)	0.21705 (5)	0.0422 (3)	
H5	0.5837	0.0503	0.1996	0.051*	
C6	0.68174 (13)	0.3111 (2)	0.19394 (5)	0.0377 (3)	
C7	0.73928 (15)	0.4729 (2)	0.21985 (5)	0.0431 (3)	
C8	0.80651 (18)	0.6541 (2)	0.19944 (5)	0.0548 (4)	
H8A	0.7337	0.7419	0.1873	0.066*	
H8B	0.8579	0.7244	0.2239	0.066*	
C9	0.92335 (19)	0.7795 (2)	0.13244 (6)	0.0612 (5)	
H9A	1.0044	0.7549	0.1141	0.073*	
H9B	0.9429	0.8954	0.1515	0.073*	
C10	0.79804 (19)	0.8230 (2)	0.09997 (6)	0.0576 (4)	
H10A	0.7165	0.8392	0.1185	0.069*	
H10B	0.8141	0.9485	0.0846	0.069*	
C11	0.76530 (15)	0.6684 (2)	0.06336 (5)	0.0446 (4)	
C12	0.67469 (14)	0.5083 (2)	0.06902 (5)	0.0410 (3)	
C13	0.60773 (14)	0.4639 (2)	0.11475 (5)	0.0411 (3)	
H13	0.6067	0.5852	0.1334	0.049*	
C14	0.68894 (14)	0.3028 (2)	0.14177 (4)	0.0390 (3)	
H14A	0.6543	0.1744	0.1314	0.047*	
H14B	0.7863	0.3110	0.1338	0.047*	
C15	1.03219 (19)	0.5291 (4)	0.18198 (7)	0.0806 (6)	
H15A	1.0963	0.5089	0.1577	0.121*	
H15B	1.0145	0.4048	0.1969	0.121*	
H15C	1.0718	0.6208	0.2042	0.121*	
C16	0.64789 (15)	0.3799 (2)	0.03173 (5)	0.0435 (3)	
H16	0.5874	0.2737	0.0354	0.052*	
C17	0.70861 (15)	0.4061 (2)	−0.01042 (5)	0.0450 (4)	
C18	0.80076 (15)	0.5652 (2)	−0.01613 (5)	0.0462 (4)	
C19	0.82693 (16)	0.6915 (2)	0.02052 (5)	0.0490 (4)	
H19	0.8882	0.7967	0.0167	0.059*	
C20	0.5989 (2)	0.1198 (3)	−0.04403 (6)	0.0634 (5)	
H20A	0.6408	0.0319	−0.0214	0.095*	
H20B	0.5888	0.0524	−0.0732	0.095*	
H20C	0.5087	0.1606	−0.0342	0.095*	
C21	0.95733 (18)	0.7328 (3)	−0.06464 (6)	0.0669 (5)	
H21A	0.9147	0.8601	−0.0605	0.100*	
H21B	0.9929	0.7246	−0.0952	0.100*	
H21C	1.0326	0.7159	−0.0421	0.100*	
N1	0.90152 (13)	0.60922 (19)	0.16244 (4)	0.0504 (3)	
O1	0.76747 (15)	0.6038 (2)	0.29889 (4)	0.0766 (4)	
O2	0.65523 (14)	0.3471 (2)	0.33582 (4)	0.0720 (4)	
O3	0.46774 (10)	0.39104 (16)	0.10842 (4)	0.0531 (3)	
H3	0.4165	0.4813	0.0992	0.080*	
O4	0.68526 (12)	0.28907 (18)	−0.04891 (3)	0.0598 (3)	
O5	0.85638 (11)	0.58148 (19)	−0.05891 (4)	0.0626 (3)	

### Atomic displacement parameters (Å^2^)

**Table:**

	*U*^11^	*U*^22^	*U*^33^	*U*^12^	*U*^13^	*U*^23^
C1	0.0611 (11)	0.0995 (15)	0.0425 (9)	0.0074 (10)	0.0007 (8)	−0.0176 (9)
C2	0.0436 (8)	0.0566 (9)	0.0436 (8)	0.0013 (7)	0.0005 (6)	−0.0111 (7)
C3	0.0411 (8)	0.0690 (10)	0.0361 (8)	0.0084 (8)	0.0029 (6)	0.0023 (7)
C4	0.0416 (8)	0.0584 (10)	0.0463 (9)	0.0007 (7)	0.0048 (6)	0.0102 (7)
C5	0.0381 (7)	0.0437 (8)	0.0448 (8)	0.0007 (6)	0.0017 (6)	−0.0003 (6)
C6	0.0329 (7)	0.0396 (8)	0.0409 (7)	0.0039 (6)	0.0043 (6)	−0.0019 (6)
C7	0.0400 (8)	0.0468 (8)	0.0430 (8)	0.0008 (6)	0.0057 (6)	−0.0055 (6)
C8	0.0655 (10)	0.0488 (9)	0.0507 (9)	−0.0124 (8)	0.0105 (8)	−0.0138 (7)
C9	0.0698 (11)	0.0517 (10)	0.0631 (11)	−0.0270 (9)	0.0159 (9)	−0.0119 (8)
C10	0.0734 (11)	0.0354 (8)	0.0653 (10)	−0.0082 (8)	0.0201 (9)	0.0018 (7)
C11	0.0473 (8)	0.0383 (8)	0.0488 (8)	0.0014 (6)	0.0082 (7)	0.0079 (6)
C12	0.0406 (8)	0.0402 (8)	0.0425 (8)	0.0027 (6)	0.0071 (6)	0.0061 (6)
C13	0.0421 (8)	0.0388 (8)	0.0431 (8)	−0.0021 (6)	0.0090 (6)	0.0005 (6)
C14	0.0413 (8)	0.0360 (7)	0.0403 (8)	−0.0014 (6)	0.0076 (6)	−0.0042 (6)
C15	0.0506 (11)	0.1104 (17)	0.0806 (14)	−0.0113 (11)	−0.0011 (9)	0.0008 (12)
C16	0.0426 (8)	0.0462 (8)	0.0420 (8)	−0.0050 (6)	0.0053 (6)	0.0063 (6)
C17	0.0411 (8)	0.0558 (9)	0.0381 (8)	0.0007 (7)	0.0018 (6)	0.0054 (7)
C18	0.0405 (8)	0.0589 (10)	0.0396 (8)	−0.0001 (7)	0.0062 (6)	0.0131 (7)
C19	0.0458 (8)	0.0465 (9)	0.0553 (9)	−0.0043 (7)	0.0082 (7)	0.0147 (7)
C20	0.0755 (12)	0.0650 (11)	0.0500 (10)	−0.0126 (9)	0.0076 (8)	−0.0057 (8)
C21	0.0538 (10)	0.0878 (13)	0.0600 (10)	−0.0096 (9)	0.0150 (8)	0.0255 (10)
N1	0.0459 (7)	0.0517 (8)	0.0541 (8)	−0.0122 (6)	0.0078 (6)	−0.0070 (6)
O1	0.0940 (10)	0.0879 (9)	0.0483 (7)	−0.0248 (8)	0.0078 (6)	−0.0235 (6)
O2	0.0812 (9)	0.0981 (10)	0.0369 (6)	−0.0062 (8)	0.0049 (6)	−0.0039 (6)
O3	0.0399 (6)	0.0642 (7)	0.0555 (7)	−0.0020 (5)	0.0074 (5)	0.0149 (5)
O4	0.0663 (7)	0.0753 (8)	0.0382 (6)	−0.0172 (6)	0.0086 (5)	−0.0013 (5)
O5	0.0566 (7)	0.0870 (9)	0.0451 (6)	−0.0144 (6)	0.0139 (5)	0.0140 (6)

### Geometric parameters (Å, º)

**Table:**

C1—O1	1.420 (2)	C12—C16	1.398 (2)
C1—O2	1.422 (2)	C12—C13	1.5200 (19)
C1—H1A	0.9700	C13—O3	1.4335 (17)
C1—H1B	0.9700	C13—C14	1.5322 (19)
C2—C3	1.374 (2)	C13—H13	0.9800
C2—C7	1.376 (2)	C14—H14A	0.9700
C2—O1	1.3793 (18)	C14—H14B	0.9700
C3—C4	1.360 (2)	C15—N1	1.457 (2)
C3—O2	1.3807 (17)	C15—H15A	0.9600
C4—C5	1.399 (2)	C15—H15B	0.9600
C4—H4	0.9300	C15—H15C	0.9600
C5—C6	1.388 (2)	C16—C17	1.3805 (19)
C5—H5	0.9300	C16—H16	0.9300
C6—C7	1.422 (2)	C17—O4	1.3745 (18)
C6—C14	1.5122 (18)	C17—C18	1.403 (2)
C7—C8	1.511 (2)	C18—O5	1.3685 (17)
C8—N1	1.4617 (19)	C18—C19	1.374 (2)
C8—H8A	0.9700	C19—H19	0.9300
C8—H8B	0.9700	C20—O4	1.420 (2)
C9—N1	1.458 (2)	C20—H20A	0.9600
C9—C10	1.524 (3)	C20—H20B	0.9600
C9—H9A	0.9700	C20—H20C	0.9600
C9—H9B	0.9700	C21—O5	1.419 (2)
C10—C11	1.509 (2)	C21—H21A	0.9600
C10—H10A	0.9700	C21—H21B	0.9600
C10—H10B	0.9700	C21—H21C	0.9600
C11—C19	1.400 (2)	O3—H3	0.8200
C11—C12	1.398 (2)		
			
O1—C1—O2	109.34 (13)	O3—C13—C14	106.08 (11)
O1—C1—H1A	109.8	C12—C13—C14	111.07 (11)
O2—C1—H1A	109.8	O3—C13—H13	109.1
O1—C1—H1B	109.8	C12—C13—H13	109.1
O2—C1—H1B	109.8	C14—C13—H13	109.1
H1A—C1—H1B	108.3	C6—C14—C13	116.11 (11)
C3—C2—C7	124.18 (14)	C6—C14—H14A	108.3
C3—C2—O1	109.97 (13)	C13—C14—H14A	108.3
C7—C2—O1	125.84 (15)	C6—C14—H14B	108.3
C4—C3—C2	121.71 (14)	C13—C14—H14B	108.3
C4—C3—O2	128.23 (15)	H14A—C14—H14B	107.4
C2—C3—O2	110.07 (14)	N1—C15—H15A	109.5
C3—C4—C5	115.72 (14)	N1—C15—H15B	109.5
C3—C4—H4	122.1	H15A—C15—H15B	109.5
C5—C4—H4	122.1	N1—C15—H15C	109.5
C6—C5—C4	123.69 (14)	H15A—C15—H15C	109.5
C6—C5—H5	118.2	H15B—C15—H15C	109.5
C4—C5—H5	118.2	C17—C16—C12	121.96 (14)
C5—C6—C7	119.32 (13)	C17—C16—H16	119.0
C5—C6—C14	119.24 (12)	C12—C16—H16	119.0
C7—C6—C14	121.44 (12)	O4—C17—C16	125.38 (14)
C2—C7—C6	115.33 (13)	O4—C17—C18	115.30 (12)
C2—C7—C8	119.32 (13)	C16—C17—C18	119.31 (14)
C6—C7—C8	125.25 (13)	O5—C18—C19	125.45 (14)
N1—C8—C7	113.84 (12)	O5—C18—C17	115.85 (14)
N1—C8—H8A	108.8	C19—C18—C17	118.70 (13)
C7—C8—H8A	108.8	C18—C19—C11	122.79 (14)
N1—C8—H8B	108.8	C18—C19—H19	118.6
C7—C8—H8B	108.8	C11—C19—H19	118.6
H8A—C8—H8B	107.7	O4—C20—H20A	109.5
N1—C9—C10	112.95 (13)	O4—C20—H20B	109.5
N1—C9—H9A	109.0	H20A—C20—H20B	109.5
C10—C9—H9A	109.0	O4—C20—H20C	109.5
N1—C9—H9B	109.0	H20A—C20—H20C	109.5
C10—C9—H9B	109.0	H20B—C20—H20C	109.5
H9A—C9—H9B	107.8	O5—C21—H21A	109.5
C11—C10—C9	115.88 (14)	O5—C21—H21B	109.5
C11—C10—H10A	108.3	H21A—C21—H21B	109.5
C9—C10—H10A	108.3	O5—C21—H21C	109.5
C11—C10—H10B	108.3	H21A—C21—H21C	109.5
C9—C10—H10B	108.3	H21B—C21—H21C	109.5
H10A—C10—H10B	107.4	C9—N1—C15	112.43 (14)
C19—C11—C12	118.27 (14)	C9—N1—C8	112.27 (13)
C19—C11—C10	117.35 (13)	C15—N1—C8	110.06 (14)
C12—C11—C10	124.35 (13)	C2—O1—C1	105.37 (14)
C16—C12—C11	118.97 (13)	C3—O2—C1	105.22 (13)
C16—C12—C13	118.44 (12)	C13—O3—H3	109.5
C11—C12—C13	122.55 (13)	C17—O4—C20	117.30 (12)
O3—C13—C12	112.35 (11)	C18—O5—C21	116.96 (13)
			
C7—C2—C3—C4	1.7 (2)	C5—C6—C14—C13	115.88 (14)
O1—C2—C3—C4	−179.25 (14)	C7—C6—C14—C13	−64.86 (17)
C7—C2—C3—O2	−178.17 (14)	O3—C13—C14—C6	−86.03 (14)
O1—C2—C3—O2	0.91 (18)	C12—C13—C14—C6	151.62 (12)
C2—C3—C4—C5	−2.1 (2)	C11—C12—C16—C17	0.2 (2)
O2—C3—C4—C5	177.69 (14)	C13—C12—C16—C17	−177.67 (13)
C3—C4—C5—C6	0.6 (2)	C12—C16—C17—O4	−178.53 (13)
C4—C5—C6—C7	1.4 (2)	C12—C16—C17—C18	0.4 (2)
C4—C5—C6—C14	−179.36 (13)	O4—C17—C18—O5	−0.76 (19)
C3—C2—C7—C6	0.4 (2)	C16—C17—C18—O5	−179.84 (13)
O1—C2—C7—C6	−178.53 (14)	O4—C17—C18—C19	178.58 (13)
C3—C2—C7—C8	176.81 (15)	C16—C17—C18—C19	−0.5 (2)
O1—C2—C7—C8	−2.1 (2)	O5—C18—C19—C11	179.15 (14)
C5—C6—C7—C2	−1.82 (19)	C17—C18—C19—C11	−0.1 (2)
C14—C6—C7—C2	178.92 (13)	C12—C11—C19—C18	0.8 (2)
C5—C6—C7—C8	−177.99 (14)	C10—C11—C19—C18	−177.30 (15)
C14—C6—C7—C8	2.8 (2)	C10—C9—N1—C15	161.30 (15)
C2—C7—C8—N1	139.97 (14)	C10—C9—N1—C8	−73.96 (17)
C6—C7—C8—N1	−44.0 (2)	C7—C8—N1—C9	158.57 (14)
N1—C9—C10—C11	−66.69 (18)	C7—C8—N1—C15	−75.39 (18)
C9—C10—C11—C19	−91.15 (17)	C3—C2—O1—C1	−1.83 (18)
C9—C10—C11—C12	90.89 (19)	C7—C2—O1—C1	177.23 (16)
C19—C11—C12—C16	−0.8 (2)	O2—C1—O1—C2	2.09 (18)
C10—C11—C12—C16	177.11 (14)	C4—C3—O2—C1	−179.40 (16)
C19—C11—C12—C13	176.99 (13)	C2—C3—O2—C1	0.42 (17)
C10—C11—C12—C13	−5.1 (2)	O1—C1—O2—C3	−1.56 (18)
C16—C12—C13—O3	−38.64 (17)	C16—C17—O4—C20	−4.4 (2)
C11—C12—C13—O3	143.52 (14)	C18—C17—O4—C20	176.55 (14)
C16—C12—C13—C14	79.99 (16)	C19—C18—O5—C21	5.0 (2)
C11—C12—C13—C14	−97.85 (15)	C17—C18—O5—C21	−175.74 (14)

### Hydrogen-bond geometry (Å, º)

**Table:**

*D*—H···*A*	*D*—H	H···*A*	*D*···*A*	*D*—H···*A*
O3—H3···O4^i^	0.82	2.31	3.0924 (16)	159
C1—H1*A*···O3^ii^	0.97	2.41	3.367 (2)	170

Symmetry codes: (i) −*x*+1, −*y*+1, −*z*; (ii) −*x*+1, *y*+1/2, −*z*+1/2.
